# Cullin Deneddylation Suppresses the Necroptotic Pathway in Cardiomyocytes

**DOI:** 10.3389/fphys.2021.690423

**Published:** 2021-06-28

**Authors:** Megan T. Lewno, Taixing Cui, Xuejun Wang

**Affiliations:** ^1^Division of Basic Biomedical Sciences, The University of South Dakota Sanford School of Medicine, Vermillion, SD, United States; ^2^Department of Cell Biology and Anatomy, University of South Carolina School of Medicine, Columbia, SC, United States

**Keywords:** Cullin, COP9 signalosome, deneddylation, necroptosis, cardiomyocyte, mice, RIPK1 signal pathway, RIPK3

## Abstract

Cardiomyocyte death in the form of apoptosis and necrosis represents a major cellular mechanism underlying cardiac pathogenesis. Recent advances in cell death research reveal that not all necrosis is accidental, but rather there are multiple forms of necrosis that are regulated. Necroptosis, the earliest identified regulated necrosis, is perhaps the most studied thus far, and potential links between necroptosis and Cullin-RING ligases (CRLs), the largest family of ubiquitin E3 ligases, have been postulated. Cullin neddylation activates the catalytic dynamic of CRLs; the reverse process, Cullin deneddylation, is performed by the COP9 signalosome holocomplex (CSN) that is formed by eight unique protein subunits, COPS1/CNS1 through COPS8/CNS8. As revealed by cardiomyocyte-restricted knockout of *Cops8* (Cops8-cko) in mice, perturbation of Cullin deneddylation in cardiomyocytes impairs not only the functioning of the ubiquitin–proteasome system (UPS) but also the autophagic–lysosomal pathway (ALP). Similar cardiac abnormalities are also observed in Cops6-cko mice; and importantly, loss of the desmosome targeting of COPS6 is recently implicated as a pathogenic factor in arrhythmogenic right ventricular dysplasia/cardiomyopathy (ARVD/C). Cops8-cko causes massive cardiomyocyte death in the form of necrosis rather than apoptosis and rapidly leads to a progressive dilated cardiomyopathy phenotype as well as drastically shortened lifespan in mice. Even a moderate downregulation of Cullin deneddylation as seen in mice with Cops8 hypomorphism exacerbates cardiac proteotoxicity induced by overexpression of misfolded proteins. More recently, it was further demonstrated that cardiomyocyte necrosis caused by Cops8-cko belongs to necroptosis and is mediated by the RIPK1–RIPK3 pathway. This article reviews these recent advances and discusses the potential links between Cullin deneddylation and the necroptotic pathways in hopes of identifying potentially new therapeutic targets for the prevention of cardiomyocyte death.

## Introduction

The ubiquitin–proteasome system (UPS) mediates the degradation of most cellular proteins that are either native or misfolded; hence, the proper functioning of the UPS is pivotal to both protein homeostasis (proteostasis) and the regulation of nearly all cellular functions. There is a large and growing body of evidence that UPS dysfunction plays a major role in cardiac pathogenesis, including the progression from a large subset of heart disease to heart failure (Wang and Wang, 2020). The latter is the leading cause of morbidity and mortality in humans. Thus, targeting UPS dysfunction is a conceivable strategy in treating heart disease, but such a strategy has not been applied clinically yet. To facilitate the development of such a new strategy, exciting progress has been made lately in research areas such as the cellular and molecular mechanisms by which cardiac UPS is regulated (Ranek et al., 2013, 2014, 2020), how to alter the regulation when needed (Zhang et al., 2019; Oeing et al., 2020; Wang and Wang, 2020), and how UPS malfunction causes cardiac injury (Tian et al., 2012; Ranek et al., 2015; Su et al., 2015), although answers to most of these questions remain incomplete.

Degradation of a substrate protein by the UPS requires polyubiquitination of the substrate protein, but the substrate specificity is conferred by ubiquitin ligases. The largest family of ubiquitin ligases is Cullin-RING ligases (CRLs), where Cullin serves as the scaffold for a RING protein and a substrate receptor module to assemble into a multiprotein complex to act as a ubiquitin ligase (Rao et al., 2020). Covalent attachment of a ubiquitin-like protein NEDD8 (Neural precursor cell Expressed Developmentally Downregulated 8) to a lysine residue of Cullin *via* a ubiquitination-like post-translational modification process known as neddylation is essential for the activation of CRLs (Li J. et al., 2020; Zhang et al., 2020). The reverse process of Cullin neddylation, referred to as Cullin deneddylation, is catalyzed by the COP9 (constitutive photomorphogenesis 9) signalosome (CSN), an evolutionally conserved protease complex (Wei and Deng, 2003; Lingaraju et al., 2014). Countering Cullin neddylation which is essential to the assembly and activation of CRLs, Cullin deneddylation triggers a timely disassembly of an active CRL that has completed ubiquitination of its specific substrate so that key components of the CRL can be recycled and used for the formation of new and different CRLs. Hence, Cullin deneddylation is equally important to the assembly/disassembly dynamic and thereby helps maintain the proper functioning of CRLs (Rao et al., 2020). Indeed, *in vivo* and genetic studies reveal that loss of function of the CSN also suppresses the overall ubiquitination activity of CRLs, although earlier *in vitro* biochemical studies suggested an inhibiting effect of Cullin deneddylation on CRLs (Wei and Deng, 2003). Mice with cardiomyocyte-restricted knockout of a canonical subunit of the CSN develop cardiomyopathy and display significantly shortened lifespans associated with massive cardiomyocyte necrosis and impairment of both the autophagic–lysosomal pathway (ALP) and UPS proteolytic function (Su et al., 2011a,b). While the CSN is ubiquitously expressed in all tissues, there seem to be tissue-specific functional roles; this review will focus on current studies on the CSN and its biological function in maintaining the survival of cardiomyocytes. Most recently, an elegant study by Liang et al. (2021) unveiled that COPS6 is a resident protein of myocardial desmosomes, a main mechanical junction of the intercalated disk. They also demonstrated that the interaction between the MPN domain of COPS6 and the spectrin repeats in the N-terminus of desmoplakin (DSP) mediates the localization of COPS6 to the desmosome. Moreover, they discovered that COPS6 protein enrichment in the intercalated disk is remarkably lost or attenuated in the myocardial biopsies from human patients with arrhythmogenic right ventricular dysplasia/cardiomyopathy (ARVD/C), a *bona fide* disease of desmosomes. Furthermore, they showed strong evidence that human ARVD/C-associated mutations in DSP and plakophilin-2 (PKP2) diminish or even abrogate the desmosome targeting of COPS6, arguing strongly for a pathogenic role of the loss of junctional COPS6 in ARVD/C; indeed, they showed that Cops6-cko recapitulates many aspects of the ARVD/C phenotype (Liang et al., 2021). This represents an important advance in cardiac CSN research because it provides not only new mechanistic insight into CSN biology but also direct evidence for the clinical relevance of investigating the CSN in the heart.

Loss of cardiomyocytes in the form of either apoptosis or necrosis can be the tipping point during the progression from various forms of primary heart disease to heart failure. Recent advances in the research into the mechanisms of cell death have unveiled that necrosis includes not only accidental but also regulated forms as well (Galluzzi et al., 2018). Depending on the primary underlying cause and thereby pathways taken, regulated necrosis can further be categorized into many forms, such as necroptosis, ferroptosis, and pyroptosis, to name a few (Galluzzi et al., 2018). Tumor necrosis factor α (TNFα) induces apoptosis in most cells; however, TNFα was found to induce necrosis in cells deficient of caspase-8 or when there was inhibition of caspases (Hitomi et al., 2008). The term necroptosis was coined to refer to this type of necrosis (Degterev et al., 2005). Hence, necroptosis is probably the earliest form of regulated necrosis described. Subsequent studies have established that the canonical pathway for death receptor activation to induce necroptosis involves the activation of the receptor-interacting protein kinase 1 (RIPK1), RIPK3, and mixed-lineage kinase domain-like pseudokinase (MLKL) (Galluzzi et al., 2018). Emerging evidence not only indicates that cardiomyocyte necroptosis plays an important role in cardiac pathogenesis but also begins unveiling the molecular mechanisms that govern cardiomyocyte necroptosis (Del Re et al., 2019). This article will review recent advances in elucidating the link between dysregulated proteostasis and necroptosis in cardiomyocytes, leveraging on the massive cardiomyocyte necrosis induced by the genetic perturbation of CSN-mediated Cullin deneddylation.

## Cullin Deneddylation by the CSN

**Figure 1:**
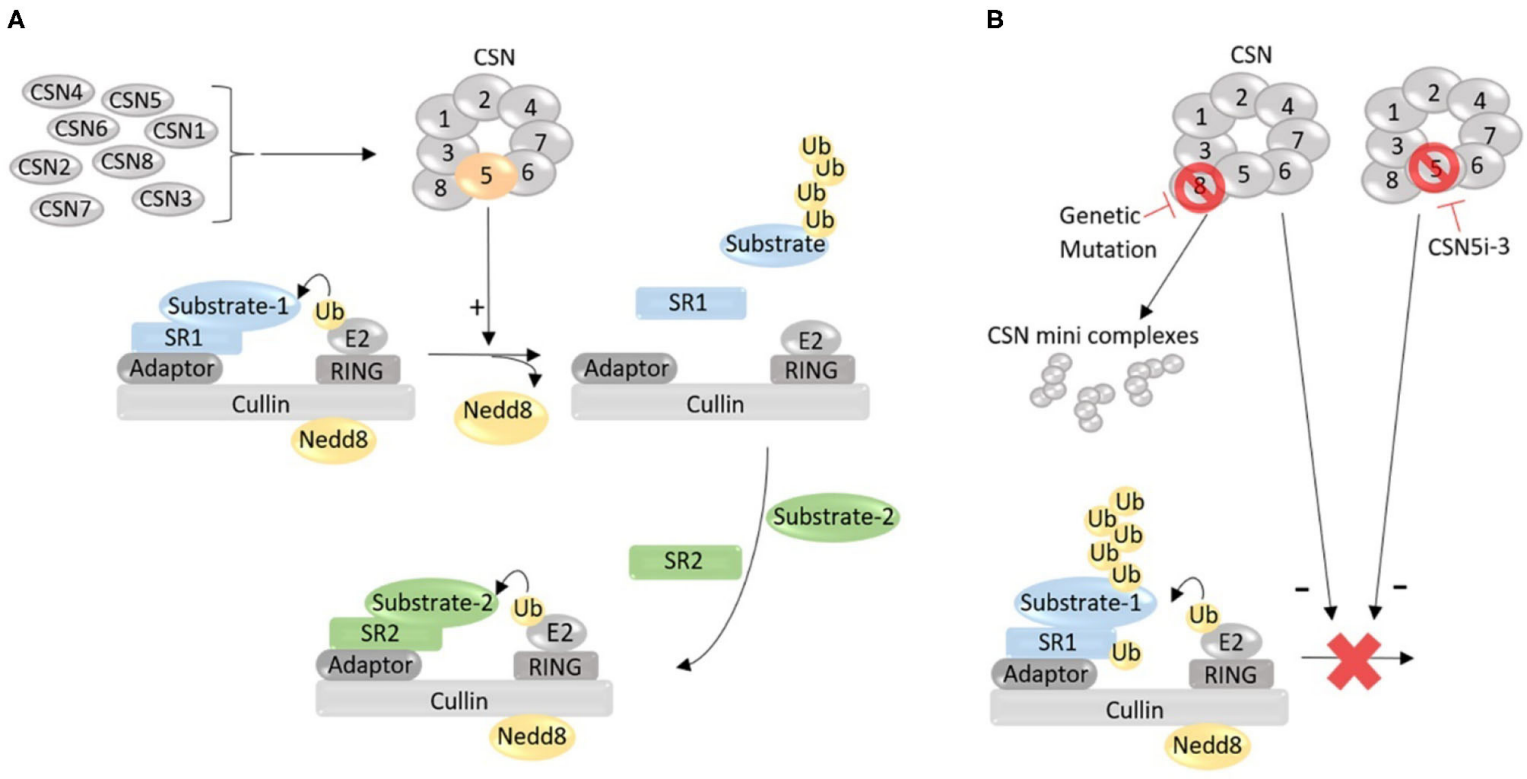
A working model for the role of the COP9 signalosome (CSN) in the regulation of the catalytic dynamic of Cullin-RING ligases (CRLs). **(A)** Under normal condition, the CSN holocomplex formed by eight unique protein subunits (CSN1 through CSN8) removes the Nedd8 from the neddylated Cullin, thereby inactivates and dissembles the CRL that has completed ubiquitinating a substrate protein (Substrate-1) recruited by substrate receptor 1 (SR1), allowing for the formation of a new CRL with a new SR (SR2) to recruit a new substrate (Substrate-2) for ubiquitination. **(B)** Defect in the formation of the CSN holocomplex, such as depletion of a subunit due to genetic mutation, may increase the abundance of certain species of minicomplexes composed of some of the CSN subunits but reduces or loses the deneddylase activity, impairing Cullin deneddylation; Cullin deneddylation by the CSN can also be inhibited by small molecules (e.g., CSN5i-3). When Cullin deneddylation is lost, the exchange of SRs in CRLs will be compromised, and as a result, the catalytic dynamic of CRLs will be stalled, leading to autoubiquitination and destruction of CRL components (e.g., SR).

Initially discovered in *Arabidopsis thaliana*, a small flowering plant (Wei et al., 1994), the CSN quickly has been found to be highly conserved in all eukaryotes, from fungi to humans (Lingaraju et al., 2014). It is composed of eight canonical subunits, which are eight unique proteins named CSN1 through CSN8 or, more officially, COPS1 through COPS8, according to the descending order of molecular weights (Kwok et al., 1998). Depletion of any of the canonical subunits impairs Cullin deneddylation, demonstrating that the deneddylase activity of the CSN requires the formation of the CSN holocomplex by the eight subunits (Figure 1). The deneddylase of the CSN resides in COPS5, but COPS5 exerts Cullin deneddylation activity only when it is situated in the fully assembled CSN holocomplex consisting of all eight subunits, which is why the loss of any of the eight CSN subunits abolishes Cullin deneddylation in the cell (Wei and Deng, 2003). A recent report claims the discovery of the ninth subunit of the CSN, a small protein with the molecular weight of 6.5 kDa, referred to as CSN acidic protein (CSNAP), but deletion of CSNAP does not alter Cullin deneddylation (Rozen et al., 2015), indicating that CSNAP is not essential to the Cullin deneddylation by the CSN. This newly discovered CSNAP might function to enhance the interaction of the CSN with CRLs (Rozen et al., 2015).

In a CRL complex, Cullin serves as a scaffold; the substrate receptor module binds to its N-terminal segment, responsible for the recruitment of a specific substrate protein for ubiquitination; and the RING protein bound to the C-terminal domain recruits the ubiquitin-charged E2. In the absence of Cullin neddylation, the substrate and the ubiquitin associated with the two arms of the CRL are too far apart for ubiquitin transfer to take place. Cullin neddylation, however, changes the conformation of the CRL so that the N- and C-terminal arms of the Cullin bend toward each other, rendering the substrate and ubiquitin-charged E2 in close proximity to each other, allowing the ubiquitin to be efficiently transferred from the E2 to the substrate protein (Rao et al., 2020). CRLs represent the largest family of E3 ubiquitin ligases. Over 700 currently known ubiquitin ligase complexes in humans belong to CRLs, responsible for the ubiquitin-dependent degradation of ~20% of cellular proteins (Dubiel et al., 2020). CRLs are crucial for UPS-mediated proteolysis of regulatory proteins and, as such, participate in the regulation of a vast array of cellular pathways and processes, such as the progression of the cell cycle. Blondelle et al. (2020) comprehensively reviewed the role of CRLs in the development, physiology, and pathology of striated muscle, including cardiac muscle.

As described earlier, neddylation is crucial for the ubiquitin ligase activity of CRLs, and disruption of neddylation *via*, for example, inhibition of NEDD8 activating enzyme 1 (NAE1) has emerged as a potential new therapeutic strategy for tumor suppression (Soucy et al., 2009; Nawrocki et al., 2013). Phase III clinical trials on neddylation inhibitor MLN4924 (also known as pevonedistat) to treat hemopoietic malignancies (NCT03268954) are ongoing. Mice with cardiomyocyte-restricted knockout of NAE1 driven by the *Myh6-Cre* displayed perinatal lethality due to myocardial hypoplasia, ventricular non-compaction, and heart failure at late gestation, which is associated with defective Hippo-YAP signaling (Zou et al., 2018). By virtue of Cullin deneddylation, the CSN modulates the assembly and activity of CRLs. Without timely Cullin deneddylation as seen in depletion of any of the canonical CSN subunits, a CRL complex cannot correctly disassemble but rather stays in its active form and continues ubiquitinating the current substrate it has bound, as well as auto-ubiquitinating its own components such as its substrate receptor, leading to self-destruction (Wee et al., 2005). Thus, loss of function of the CSN can impair the overall ubiquitination by CRLs by hindering the exchange of substrate receptors in CRLs (Lydeard et al., 2013; Rao et al., 2020). A small molecule inhibitor of the CSN (CSN5i-3) has been reported to impair Cullin deneddylation and shows great promise in the experimental treatment of cancer (Schlierf et al., 2016).

## Pathways to Cardiomyocyte Necroptosis

Necroptosis is a form of caspase-independent regulated cell death that shares the morphologic characteristic of necrosis. The prototype of necroptosis is the necrosis induced by stimulation of the death receptor family in cells deficient of intracellular apoptotic signaling (Degterev et al., 2005). It is characterized by the lack of the typical nuclear condensation and internucleosomal DNA fragmentation that are characteristic of apoptosis and by the presence of enlarged and swollen organelles and early cell plasma membrane perforation and resultant release of inflammatory damage-associated molecular patterns (DAMPs), which initiate innate immune responses, resulting in an inflammatory response (Zhou and Yuan, 2014; Pasparakis and Vandenabeele, 2015; Wegner et al., 2017; Samson et al., 2021). Consequently, necroptosis has been widely implicated in innate immunity and inflammatory diseases (Zhou and Yuan, 2014; Pasparakis and Vandenabeele, 2015; Wegner et al., 2017; Molnar et al., 2019).

For detection of apoptosis, a well-defined set of assays have been developed on the basis of its unique biochemical and morphological features, but the current situation for the identification of necroptosis or any forms of regulated necrosis is quite different because all regulated necrosis [e.g., necroptosis, ferroptosis, pyroptosis, and MPT (mitochondrial permeability transition)] and even incidental necrosis share virtually the same morphological changes although the molecular pathways to the various forms of regulated necrosis differ (Del Re et al., 2019). Thus, the detection of these forms of necrosis must rely on a combination of assessments that identify the co-existence of necrosis with the activation of the mechanistic pathway characteristic of the respective forms of regulated necrosis (Mishra et al., 2019). For cardiomyocyte necroptosis, the following pathways have been documented.

### The RIPK1–RIPK3–MLKL Pathway

**Figure 2:**
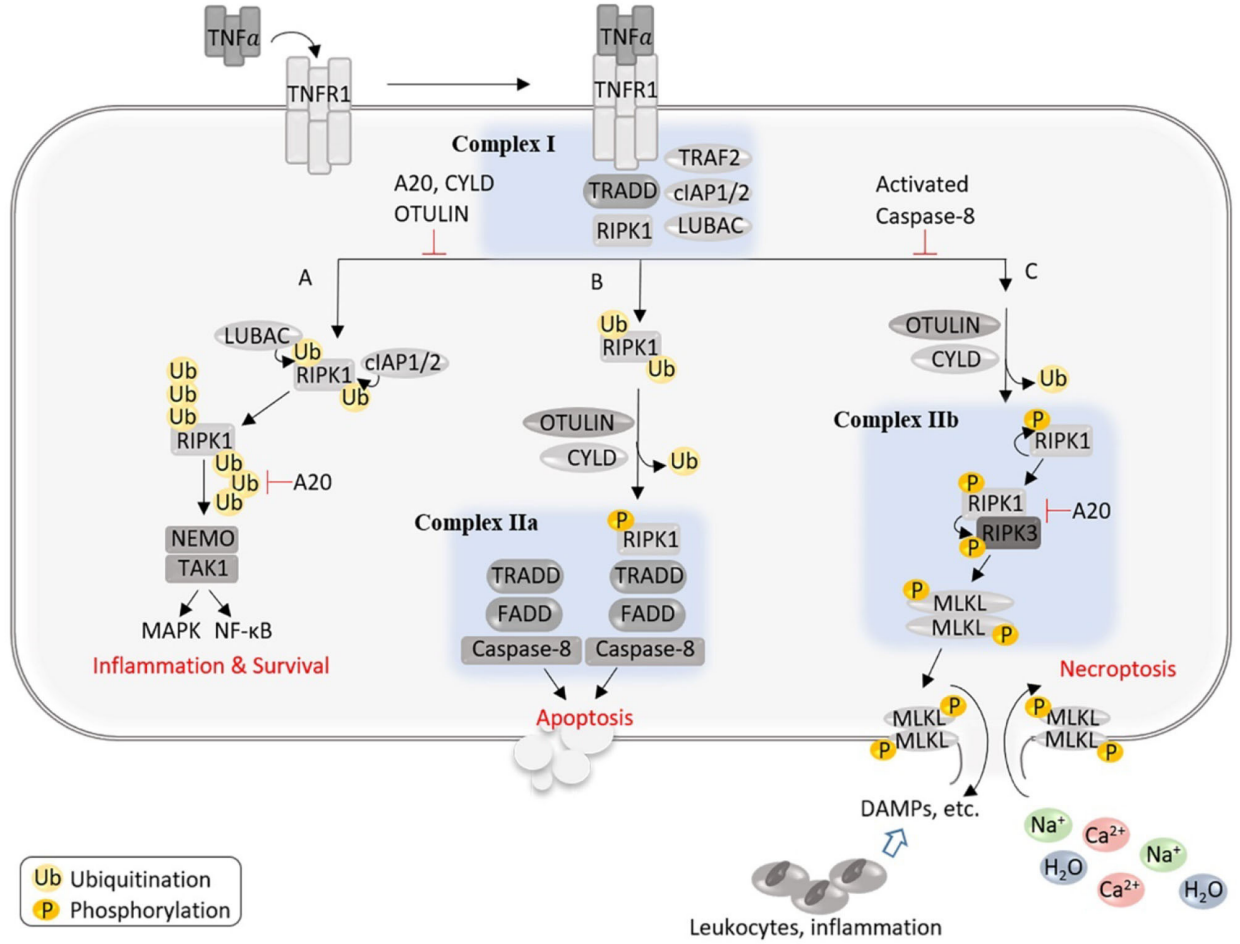
An illustration of the potential signaling events and outcomes after the activation of TNFR1. The binding of TNFα to TNFR1 induces the formation of a membrane-bound assembly known as Complex I, which is associated with the cytoplasmic tail of TNFR1 and contains TRADD, RIPK1, TRAF2, LUBAC, and cIAP1/2, where LUBAC adds linear ubiquitin chains to RIPK1 and other proteins, whereas cIAP1/2 catalyze the addition of K63-linked polyubiquitin chains to RIPK1 and other proteins within Complex I. The ubiquitinated RIPK1 functions as a scaffold to recruit NEMO and TAK1, leading to the activation of NFκB and MAPK pathways, respectively, and promotes cell survival and inflammation **(A)**. Complex I can be transformed into the secondary cytosolic Complex IIa and IIb to signal apoptosis **(B)** or necroptosis **(C)**, respectively. **(B)** Caspase-8 activity in Complex IIa determines downstream signaling toward an apoptotic outcome and simultaneously prevents necroptosis by cleaving RIPK1 or RIPK3. **(C)** In Complex IIb, the absence or inactivation of caspase-8 triggers autophosphorylation of RIPK1, which in turn binds and phosphorylates RIPK3; phosphorylated RIPK3 recruits and phosphorylates MLKL, leading to MLKL's translocation to the plasma membrane where MLKL oligomerizes and forms pores, allowing an influx of extracellular ions and water into the cell that causes cell swelling and rupture, and the release of cellular contents triggers proinflammatory responses.

At the molecular level, necroptosis can be induced by activation of death receptors upon engagement of their respective ligands, which includes binding of TNFα to tumor necrosis factor receptor 1 (TNFR1), first apoptotic signal ligand (FasL) to Fas, TNF-related apoptosis-inducing ligand (TRAIL) to TRAIL receptor 1/2 (TRAIL-R1/2), interferons (INFs) to INF receptors (INFRs), Toll-like receptor (TLR) ligands to TLR3/4, or by binding of virus Z-form DNA or RNA to the cytosolic nucleic acid sensor, Z-DNA binding protein 1 (ZBP1) (Zhou and Yuan, 2014; Pasparakis and Vandenabeele, 2015; Wegner et al., 2017; Samson et al., 2021). Necroptosis triggered by TNFα ligation of TNFR1 is best understood (Figure 2), thus being considered as the prototypical form (Zhou and Yuan, 2014). In this model, the binding of TNFα to TNFR1 induces the recruitment of various proteins to the cytoplasmic tail of TNFR1, including TNFR-associated death domain (TRADD), RIPK1, TNFR-associated factor 2 (TRAF2), linear ubiquitin chain assembly complex (LUBAC), cellular inhibitor of apoptosis-1 (cIAP1), and cIAP2, forming a membrane-bound assembly known as Complex I (Zhou and Yuan, 2014; Pasparakis and Vandenabeele, 2015; Wegner et al., 2017; Samson et al., 2021). At this point, LUBAC adds N-terminal methionine (Met1)-linked linear ubiquitin chains to RIPK1 and other proteins, whereas cIAP1/2 catalyze the addition of K63-linked polyubiquitin chains to RIPK1 and other proteins within Complex I. The ubiquitinated RIPK1 functions as a scaffold to recruit nuclear factor κB (NFκB) essential modulator (NEMO, also known as an inhibitor of NFκB kinase subunit gamma, IKKγ) and transforming growth factor beta-activating kinase 1 (TAK1), leading to the activation of NFκB and mitogen-activated protein kinase (MAPK) pathways, respectively (Figure 2). Activation of these pathways promotes the synthesis of proinflammatory cytokines and cell survival. Of note, RIPK1 mediates NFκB activation independent of its kinase activity. However, Complex I can be transformed into the secondary cytosolic complex, Complex II, which mediates cell death. This transition requires deubiquitination of Complex I and association with Fas-associated protein with death domain (FADD) and caspase-8. Complex II may contain different components and promote cell death in a manner that is either independent or dependent of the kinase activity of RIPK1. The canonical RIPK1 kinase-independent proapoptotic caspase-8 complex is termed Complex IIa (also known as ripoptosome), whereas RIPK1 kinase-dependent complexes are named Complex IIb (also named necrosome). Complex IIa may be composed of TRADD, FADD, RIPK1, or RIPK3, while Complex IIb consists of RIPK1, RIPK3, MLKL, FADD, and inactive caspase-8, although the complete composition of these complexes is not yet established. Caspase-8 activity in Complex IIa determines downstream signaling toward an apoptotic outcome by activating Bid and caspase-3 and simultaneously prevents necroptosis by cleaving RIPK1 or RIPK3. Inactivation of caspase-8 in Complex IIb promotes necroptosis *via* a core signaling axis of RIPK1–RIPK3–MLKL, which is called canonical necroptosis. In the canonical pathway, blockade of caspase activity triggers autophosphorylation of RIPK1 on Ser166 (Degterev et al., 2008), which in turn assembles with RIPK3 into hetero-amyloid filaments, resulting in RIPK3 phosphorylation on Ser232 as well as subsequent recruitment of downstream necroptosis effector, MLKL, and phosphorylating MLKL on Thr357/Ser358 sites (Sun et al., 2012). This RIPK3-mediated phosphorylation of MLKL triggers a conformational change in MLKL, leading to its translocation to the plasma membrane, where it oligomerizes and forms pores that mediate necroptosis by lysing the membrane (Dondelinger et al., 2014; Wang et al., 2014). Necroptotic signaling triggered by the activation of other receptors upon engagement of their ligands may be different at the upstream, but all converge with RIPK3-dependent phosphorylation and activation of MLKL toward plasma membrane lysis (Pasparakis and Vandenabeele, 2015; Wegner et al., 2017; Samson et al., 2021).

It should be noted that signaling mechanisms underlying necroptosis act in a context-, cell type-, and species-dependent manner and are still far from a comprehensive understanding (Wegner et al., 2017; Samson et al., 2021). Physiologically, the essential regulators which determine activation of distinct death pathways remain unclear. In addition, the endogenous factors which determine the formation of Complex IIb (necrosome) are not well-understood. Intriguingly, a few deubiquitinating enzymes (DUBs), such as cylindromatosis (CYLD), A20 (Tnfaip3), and OTULIN (ovarian tumor deubiquitinase with linear linkage specificity), appear to regulate necrosome formation and outcome. CYLD deubiquitinates RIPK1 to decrease the interaction of RIPK1 with NEMO, thus destabilizing Complex I, whereas it also deubiquitinates RIPK1 in TNFα-induced necrosome to facilitate kinase activation and necroptosis (O'Donnell et al., 2011; Moquin et al., 2013). CYLD is negatively regulated by caspase-8-mediated cleavage, protecting against TLR-mediated necroptosis (Legarda et al., 2016). The ubiquitin-chain editing function of A20 can replace K63 polyubiquitin chains from RIPK1 with K48 polyubiquitin chains, leading to its proteasomal degradation (Wertz et al., 2004), while restricting K63-ubiquitination of RIPK3 at K5 and reducing its binding to RIPK1, thus suppressing necroptosis (Onizawa et al., 2015). Mutations causing hypomorphic A20 expression and function are linked to a wide range of human inflammatory and autoimmune diseases (Martens and van Loo, 2020), and as necroptosis is intimately involved in inflammation, a better understanding of how A20 regulates necroptosis is expected to advance the investigation into the pathogenesis of human inflammatory and autoimmune disease. In addition, OTULIN inhibits necroptosis *via* downregulation of necroptotic RIPK1 ubiquitination and activation (Douglas and Saleh, 2019). Cell-type-specific knockout approaches demonstrate non-redundant functions of these DUBs, which are presumably explained by their differential specificity for different types of ubiquitin chains (Lork et al., 2017). However, the molecular mechanisms underlying specific functions of CYLD, A20, and OUTLIN as well as their crosstalk with each other or with other DUBs remain poorly understood.

The complexity of necroptosis regulation is further intensified by emerging crosstalk between necroptosis and other types of necrotic cell death, including pyroptosis, ferroptosis, and mitochondrial permeability transition pore (MPT)-mediated necrosis. Unlike the reciprocally negative regulation between apoptosis and necroptosis, these types of necrotic cell death usually help each other in facilitating necrotic processes *via* yet unknown mechanisms (Kist and Vucic, 2021). In addition, necroptosis is usually associated with the accumulation of autophagosomes, double membrane-enclosed vesicles that package cytoplasmic components and deliver the cargo to lysosomes for degradation (Shen and Codogno, 2012). As discussed specifically in the *Interactions between macroautophagy and the RIPK1–RIPK3–MLKL pathways* section, autophagy may function as a prosurvival mechanism *via* suppression of necroptosis.

### The RIPK3–CaMK2–MPT Pathway

An early study showed that RIPK3 was upregulated in ischemic myocardium, but the infarct size of a myocardial infarction (MI) model induced by a comparable permanent ligation of the coronary artery did not seem to differ between wild-type (WT) and RIPK3^−/−^ mice (Luedde et al., 2014). Nevertheless, this study has provided the first experimental evidence that RIPK3, a critical kinase regulating necroptosis, plays a mediator role in post-MI maladaptive remodeling. This is because this study revealed that myocardial reactive oxygen species (ROS) levels examined at 24 h post-MI, the CD3-positive cell infiltration examined at 4 days post-MI, and the maladaptive cardiac remodeling observed at 30 days post-MI were all significantly attenuated in the RIPK3^−/−^ mice compared with WT mice (Luedde et al., 2014). Furthermore, data collected from neonatal rat ventricular myocyte (NRVM) cultures did support the requirement of RIPK3 and potentially its interaction with RIPK1 for the induction of cardiomyocyte necrosis by treatment of TNFα combined with a broad-spectrum caspase inhibitor (zVAD-fmk) (Luedde et al., 2014), but no direct evidence was provided for the role of cardiomyocyte necroptosis in post-MI remodeling. This is because RIPK3 was ablated in all cells in RIPK3^−/−^ mice, and the protection of RIPK3 deficiency could have come from the reduced inflammatory responses resulting from the loss of RIPK3 in inflammatory cells.

A subsequent study using myocardial ischemia–reperfusion (I-R) injury and doxorubicin (Dox)-induced acute cardiotoxicity models showed that cardiomyocyte necrosis was significantly reduced in RIPK3^−/−^ mice compared with WT mice (Zhang et al., 2016), indicating that RIPK3 activation plays an essential role in I-R injury and Dox cardiotoxicity. Moreover, these authors presented evidence that RIPK3 does so through activation of calcium-calmodulin-dependent protein kinase II (CaMKII) rather than through the well-established partners RIPK1 and MLKL (Zhang et al., 2016). They showed that siRNA-mediated knockdown of either RIPK1 or MLKL yielded no significant effects on the leakage of LDH and the reduction of cell viability induced by adenovirus-mediated RIPK3 overexpression in cultured NRVMs. The increases of myocardial Thr287-phosphorylated CaMKII in response to I-R injury or Dox treatment were attenuated or abolished by RIPK3 deficiency in mice, and RIPK3 was found to bind to and, *via* both phosphorylation and oxidation, activate CaMKII in cultured NRVMs (Zhang et al., 2016). It is well-known that CaMKII is activated and plays a mediating role in myocardial cell death and I-R injury (Bell et al., 2014). Hence, it is not surprising that treatment of a CaMKII inhibitor (KN-93) was found to effectively reduce the infarct size and serum LDH elevation induced by myocardial I-R and similarly attenuate the Dox-induced cardiac damage and malfunction in WT mice (Zhang et al., 2016). Importantly, adenoviral overexpression of a dominant-negative CaMKII was shown to reduce the cytotoxicity induced by not only Dox treatment or hypoxia/reoxygenation (H/R) but also RIPK3 overexpression in NRVM cultures (Zhang et al., 2016). It should be noted that this study has also unveiled that RIPK3 contributes to the induction of apoptosis by myocardial I-R, and CaMKII plays a mediating role in the caspase activation by RIPK3 overexpression in cultured NRVMs. Hence, the RIPK3–CaMKII pathway appears to be responsible for both necroptosis and apoptosis in I-R injury and acute Dox cardiotoxicity.

**Figure 3:**
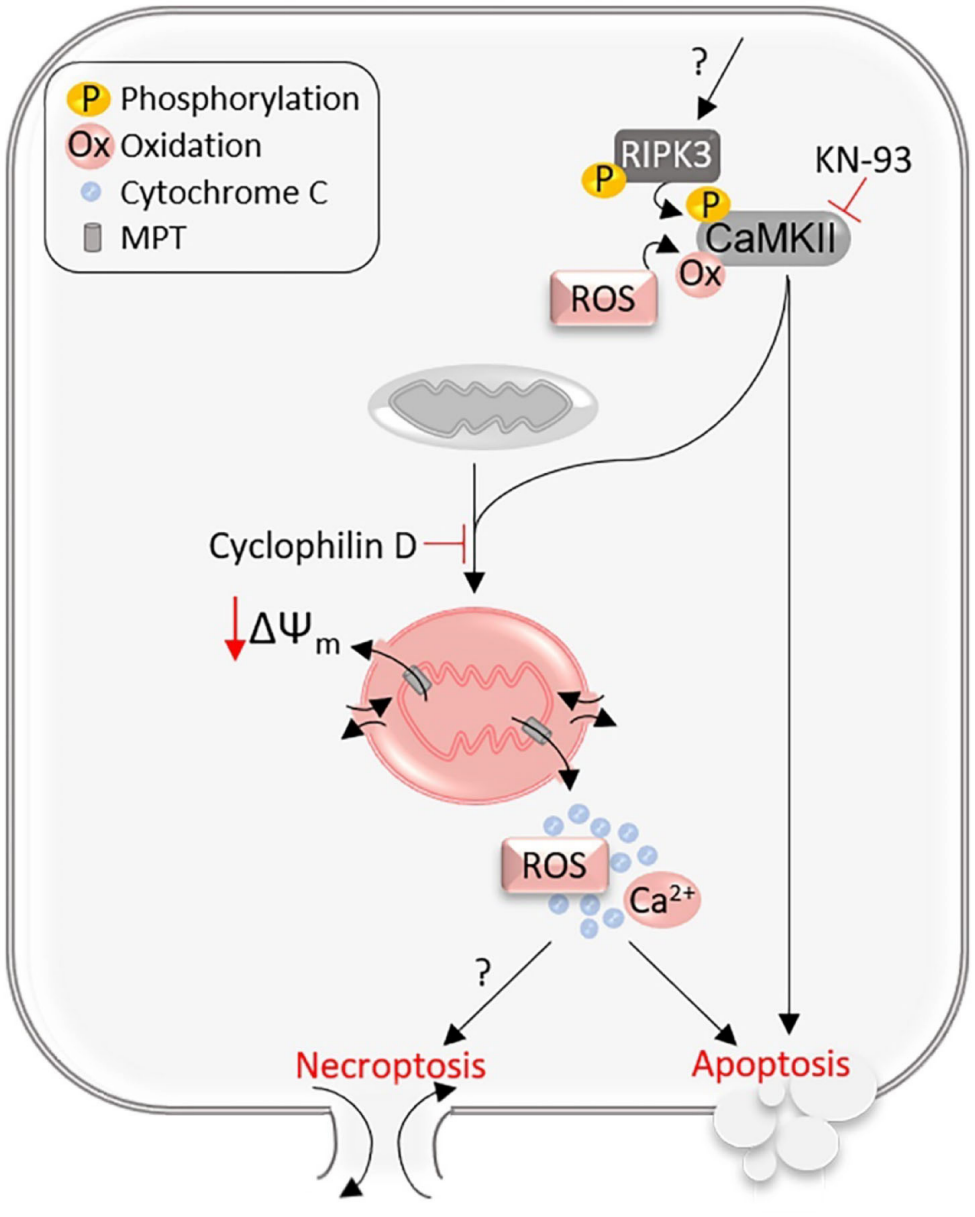
The RIPK3–CaMKII–MPT pathway. RIPK3 phosphorylated and activated through an unknown mechanism binds to and phosphorylates CaMKII. Both phosphorylation and oxidation on CaMKII can activate CaMKII, which opens the mitochondrial membrane permeability transition (MPT) pores, inducing mitochondrial membrane depolarization (ΔΨ_m_) and release of mitochondrial contents, and thereby the RIPK3–CaMKII–MPT pathway mediates both necroptosis and apoptosis in cardiomyocytes.

Since the activation of CaMKII was known to cause cardiomyocyte death *via* opening MPT pores (Joiner et al., 2012), Zhang et al. further tested whether MPT is the downstream executor of the RIPK3–CaMKII pathway in NRVM cultures. They found that siRNA-mediated downregulation of cyclophilin D, a key regulator of MPT, moderately but statistically significantly alleviated RIPK3 overexpression-induced cell death as assessed by LDH release and cell viability assays (Zhang et al., 2016). It was also found that RIPK3 deficiency reduced the induction of the depolarization of the mitochondrial membrane potential (ΔΨ_m_) by I-R or Dox treatment, RIPK3 overexpression induced ΔΨ_m_ depolarization in a cyclophilin D-dependent manner, and inhibition of CaMKII prevented RIPK3 from inducing ΔΨ_m_ depolarization (Zhang et al., 2016). Taken together, these *in vivo* and *in vitro* findings support a RIPK3–CaMKII–MPT pathway to cardiomyocyte necroptosis (Figure 3). This blurs the boundary between necroptosis and the MPT-driven necrosis (Galluzzi et al., 2018); the latter can be differentiated from other types of regulated necrosis by its dependency on cyclophilin D (Baines et al., 2005), according to a recent classification scheme (Galluzzi et al., 2018). The RIPK3–CamKII–MPT pathway may be unique to cardiomyocytes because a prior study demonstrated that cells depleted of mitochondria can still undergo necroptosis (Tait et al., 2013), arguing against an obligatory role of MPT opening in necroptosis. The RIPK1/RIPK3-mediated necroptosis and MPT-driven necrosis have been shown to contribute uniquely to renal I-R injury (Linkermann et al., 2013), whereas RIPK1 and MPT were implicated to work in the same pathway in myocardial I-R injury (Lim et al., 2007).

The defining evidence provided by Zhang et al. (2016) to support the role of MPT opening in the RIPK3–CaMKII necroptotic pathway was collected primarily from cell cultures, which may represent a caveat. This is because in the cell culture setting, a cell undergoing apoptosis may also show loss of its membrane integrity (the characteristic of necrosis), although this does not occur *in vivo* (Del Re et al., 2019). Therefore, decreased cell viability and increased LDH leakage induced by RIPK3 overexpression in cultured NRVMs might not necessarily result from necroptosis, especially when RIPK3 can mediate both necroptosis and apoptosis as shown by this and other prior studies. It also should be pointed out that evidence for ruling out the involvement of MLKL or RIPK1 also seems to be quite weak, and further studies to gather more comprehensive and especially *in vivo* genetic evidence are warranted. MLKL knockout mice have been reported and used to test the involvement of MLKL in necroptosis and pathogenesis (Wu et al., 2013); it will be very interesting and important to test whether MLKL null mice show resistance to I-R injury and Dox cardiotoxicity. As highlighted in an earlier section, the requirement of RIPK1 in cardiomyocyte necroptosis induced by at least TNFR1 signaling has been well-demonstrated.

## Interactions Between Macroautophagy and the RIPK1–RIPK3–MLKL Pathways

Emerging evidence increasingly suggests that the processes of autophagy and necroptosis interact at multiple levels in both non-myocyte cells and cardiomyocytes. For example, lysosome inhibition with bafilomycin A1 is capable of accumulating RIPK1 and RIPK3, and conversely, rapamycin-activated autophagy protects against necroptosis in rat pheochromocytoma PC12 cells (Liu et al., 2018). ATG16L-dependent autophagy has been shown to clear RIPK1, PIPK3, TRIF, and ZBP1 and attenuate necroptosis induced by TNFα and TLR ligands in macrophages (Lim et al., 2019). This is further complicated by the observations that MLKL suppresses autophagy, most likely at the level of autolysosome efflux, while the protein turnover of MLKL is indeed controlled by autophagy in hepatocytes under physiological conditions (Wu et al., 2020b). Lipid overloading intensifies MLKL-mediated autophagy inhibition and necroptosis in hepatocytes (Wu et al., 2020b). In addition, although MLKL attenuates autophagy characterized by autophagosome and autolysosome dysfunction in immortalized mouse dermal fibroblasts and HT-29 colorectal cancer cells treated with TSI (TNF, the SMAC mimetic Compound A, and caspase inhibitor IDN-6556), suppression of autophagosome formation *via* CRISPR/Cas9-mediated knockout of autophagy-related gene 5 (Atg5) or of Atg7 does not affect necroptosis in these cells (Frank et al., 2019). Taken together, these findings from non-cardiomyocytes seem to indicate that defective removal of autophagosomes resulting from decreased autophagic flux may contribute to necroptosis.

At the molecular level, autophagy-initiating kinase ULK1 has been shown to phosphorylate RIPK1 at Ser357, thereby inhibiting TNF-induced cell death (Wu et al., 2020a). Activation of mTOR is known to suppress autophagy. A recent study showed that mTOR hyperactivation by either Western diet or genetic inhibition of Tsc1 led to necroptosis of gut epithelia, contributing to inflammatory bowel disease; mechanistically, mTOR suppresses TRIMM11-mediated ubiquitination of RIPK3, and the latter targets RIPK3 for autophagic degradation (Xie et al., 2020). A different group subsequently reported that during necroptosis, RIPK3 reduces autophagosome–lysosome fusion and thereby decreasing autophagic flux in intestine epithelia (Otsubo et al., 2020). MLKL-dependent but RIPK3-independent signaling was shown to suppress autophagy in hepatocytes, contributing to Western diet-induced liver injury in mice (Wu et al., 2020b). In other cells undergoing necroptosis, the association of active MLKL with cell membrane was shown to suppress autophagosome removal (Frank et al., 2019). Conversely, the phosphorylation of ULK1 at Ser746 by RIPK3 was shown to be required for the induction of alternative autophagy by genotoxic stress (Torii et al., 2020).

It appears that impaired autophagic flux contributes to necroptosis in cardiomyocytes. Two recent reports showed evidence collected from cultured H9c2 cells suggesting a major contribution of impaired autophagy to the induction of necroptosis by TNFα (Ogasawara et al., 2017; Abe et al., 2019). According to these reports, autophagic flux was suppressed as the RIPK1–RIPK3 interaction and necroptosis were induced by the combined treatment with TNFα and a broad-spectrum caspase inhibitor (Ogasawara et al., 2017); improving autophagic flux through inhibition of mTORC1 was able to attenuate the necroptosis in an autophagy- and transcription factor EB (TFEB; a master regulator of the ALP)-dependent fashion (Ogasawara et al., 2017; Abe et al., 2019); and MPT was not important in the execution of necroptosis (Ogasawara et al., 2017). More recently, Li C. et al. (2020) reported that aging-associated impairment of autophagy promoted myocardial I-R injury, the protection of metformin against such injury was associated with improving autophagic flux, and the upregulation of p62 resulting from decreased autophagy promoted the interaction of RIPK1 and RIPK3, a key step for the activation of the RIPK1–RIPK3-mediated necroptotic pathway. Given that both necroptosis and autophagic impairment have been shown as mediators in cardiac pathogenesis, it will be extremely important to improve our understanding of the interaction between autophagy and necroptosis in cardiomyocytes as the resultant mechanistic insight is expected to help identify potentially new therapeutic strategies to treat a large subset of heart disease.

## Cardiac Cops8 Deficiency Causes Massive Cardiomyocyte Necrosis and Heart Failure in Mice

The cardiac physiological significance of the CSN has been studied using mice with cardiomyocyte-restricted Cops8 knockout (Cops8-cko) (Su et al., 2011a,b). In mice with Cops8-cko produced by coupling a Cops8-floxed allele with a transgenic *Cre* driven by the mouse α myosin heavy chain (*Myh6*) promoter, the depletion of Cops8 protein in cardiomyocytes was found to take place during the perinatal period; hence, this Cops8-cko is referred to as perinatal Cops8-cko. The perinatal Cops8-cko did not appear to affect prenatal cardiac development but remarkably affected post-natal cardiac development and functioning. In these mice, pathological cardiac hypertrophy was evident at 2 weeks post-natal, left ventricular (LV) chamber dilatation and malfunction became detectable by 3 weeks, and decompensated left heart failure (as reflected by increased lung weight to body weight ratios) as well as overt development retardation (as reflected by lack of body weight gain between 3 and 4 weeks) was discerned between 3 and 4 weeks of age. Mice with perinatal Cops8-cko all died by post-natal 52 days, with a median lifespan of ~32 days (Su et al., 2011b). These findings demonstrate that Cops8 is indispensable for post-natal cardiac development and cardiac function. Since the Cops8-cko mice display remarkably reduced myocardial protein levels of virtually all other CSN subunits and increases in neddylated Cullins as expected, the abnormal phenotype observed in the Cops8-cko mice is conceivably attributable to perturbation of the CSN and of Cullin deneddylation although this remains to be confirmed by conditional knockout of at least another CSN subunit.

Biochemical and histopathological examination by Su et al. (2011b) revealed that cardiomyocyte apoptosis in Cops8-cko mice was not increased until 4 weeks of age when overt heart failure had occurred; moreover, cardiac overexpression of anti-apoptotic protein BCL2 failed to delay the premature death of Cops8-cko mice. These findings compellingly indicate that apoptosis is not a primary cause of the cardiac pathology induced by Cops8-cko. Further examination revealed that massive cardiomyocyte necrosis took place in the Cops8-cko mice as early as 3 weeks of age, as evidenced by increased *in vivo* Evans blue dye (EBD) uptake by cardiomyocytes, increased leukocyte infiltration, and necrotic morphology detected by transmission electron microscopy (Su et al., 2011a,b). Furthermore, massive cardiomyocyte necrosis, dilated cardiomyopathy, rapidly progressed heart failure, and an overwhelmingly increased mortality were subsequently observed in Cops8-cko initiated in adult mice as well (Su et al., 2013), indicating that post-natal cardiac development-associated cardiomyocyte proliferation and hypertrophy are not a required compounding factor for the induction of cardiomyocyte necrosis by Cops8 deficiency. Notably, apoptosis appears to be the primary mode of cell death observed in mice with conditional deletion of the Cops8 in peripheral T lymphocytes and in the liver of mice with the hepatocyte-restricted ablation of *Cops8* (Menon et al., 2007; Lei et al., 2011, 2013); hence, the mode of cell death induced by Cops8/CSN deficiency may be tissue- or cell-type specific. It will be interesting to dissect the mechanism governing this specificity.

## Mechanisms Underlying Cardiomyocyte Necrosis Induced by Cops8 Deficiency

### The Cardiomyocyte Necrosis in Cops8-cko Mice Is Primarily RIPK1–RIPK3-Mediated Necroptosis

To determine the nature of the cardiomyocyte necrosis induced by Cops8-cko, Xiao et al. (2020) examined the potential involvement of the RIPK1–RIPK3 pathway. They found that myocardial protein levels of major known players of the canonic necroptotic pathway (RIPK1, RIPK3, RIPK1-bound RIPK3, and MIKL) were all markedly increased in the face of significantly increased myocardial Bcl2 and decreased cleavage of caspase-8 in mice with perinatal Cops8-cko (Xiao et al., 2020). These findings are indicative of suppressed apoptotic pathways but an activation of the RIPK1–RIPK3–MLKL pathway in Cops8-cko mouse hearts. This is because defective caspase-8 or suppression of the apoptotic pathway has been shown as the prerequisite for death receptor activation to induce necroptosis, and the increased binding of RIPK3 with RIPK1 is a hallmark of the activation of the necroptotic pathway by death receptor stimulation (Cho et al., 2009; He et al., 2009; Zhang et al., 2009). To further establish the role of the RIPK1–RIPK3 pathway, Xiao et al. (2020) tested the effects of necrostatin-1 (Nec-1, a specific kinase inhibitor of RIPK1) and of germline RIPK3 knockout on the cardiomyocyte necrosis and mouse premature death induced by Cops8-cko. The osmotic minipump-mediated treatment of the Cops8-cko mice with Nec-1 initiated at 2 weeks of age achieved a nearly complete blockade of cardiomyocyte necrosis examined at 3 weeks of age and significantly delayed mouse premature death, demonstrating that kinase activity of RIPK1 is required for the induction of cardiomyocyte necrosis by Cops8-cko. Moreover, heterozygous knockout of *Ripk3* also significantly attenuated cardiomyocyte necrosis and elongated the lifespan of mice with Cops8-cko (Xiao et al., 2020). Taken together, these experimental findings provide unequivocal evidence that cardiomyocyte necrosis induced by Cops8-cko is primarily necroptosis mediated by the RIPK1–RIPK3 pathway. The upregulation of myocardial MLKL proteins in Cops8-cko mice suggests that MLKL is probably the downstream effector for this pathway, but this remains to be established because neither the phosphorylation status of MLKL nor the requirement of MLKL in the increased necrosis has been examined in these mice.

### Cardiomyocyte Necroptosis Induced by Cops8-cko Is Independent of MPT

As discussed in the *Pathways to cardiomyocyte necroptosis* section, MPT-dependent necrosis by itself can be an independent form of regulated necrosis (Galluzzi et al., 2018); more recently, a RIPK3–CamKII–MPT pathway to cardiomyocyte necroptosis was reported to play an important role in myocardial I-R injury and acute Dox cardiotoxicity (Zhang et al., 2016). Cyclophilin D is a mitochondrial peptidyl-prolyl cis-trans isomerase, a genetically confirmed activator of MPT, and is required for MPT (Baines et al., 2005). Hence, the golden standard for the identification of MPT-dependent necrosis is to show that the necrosis can be blocked by depletion of cyclophilin D (Galluzzi et al., 2018). Neither homozygous nor heterozygous knockout of cyclophilin D was able to attenuate cardiomyocyte necrosis or premature death of Cops8-cko mice (Xiao et al., 2020), demonstrating that cardiomyocyte necrosis induced by Cops8-cko is independent of MPT. Intriguingly, depletion of cyclophilin D *via* homozygous knockout exacerbated cardiomyocyte necrosis and shortened the lifespan of Cops8-cko mice, suggesting that homeostatic levels of cyclophilin D are essential for cardiomyocyte survival and heart function under the stress condition created by Cops8-cko. This is in agreement with a prior report showing that cyclophilin D knockout increases the propensity for heart failure in mice (Elrod et al., 2010).

### How Could Cops8 Deficiency Activate the Cardiac RIPK1–RIPK3 Necroptotic Pathway?

Although the link between Cops8/CSN deficiency and activation of the RIPK1–RIPK3 necroptotic pathway has not been delineated, existing literature has offered possible candidates. First of all, increases of p62 and LC3-II proteins and decreased autophagic flux due to impaired autophagosome–lysosome fusion occur in the cardiomyocytes of mice with perinatal Cops8-cko before 2 weeks of age, which is clearly before cardiomyocyte necrosis becomes detectable (Su et al., 2011a); hence, autophagic impairment, especially combined with the UPS dysfunction that results directly from Cops8 deficiency (Su et al., 2015) and indirectly from autophagic impairment (Tian et al., 2014; Wang and Wang, 2015), could have served as an underlying cause of the cardiomyocyte necrosis *via* a RIPK1–RIPK3-dependent or -independent pathway. This is because it has been shown that duo inhibition of the proteasome and autophagy is sufficient to cause cardiomyocyte necrosis in mice (Su et al., 2011a) and, as discussed in the *Interactions between macroautophagy and the RIPK1–RIPK3–MLKL pathways* section, accumulated p62 may serve as a scaffold to promote the interaction between RIPK1 and RIPK3 and thereby activation of the RIPK1–RIPK3 pathway (Li C. et al., 2020).

**Figure 4:**
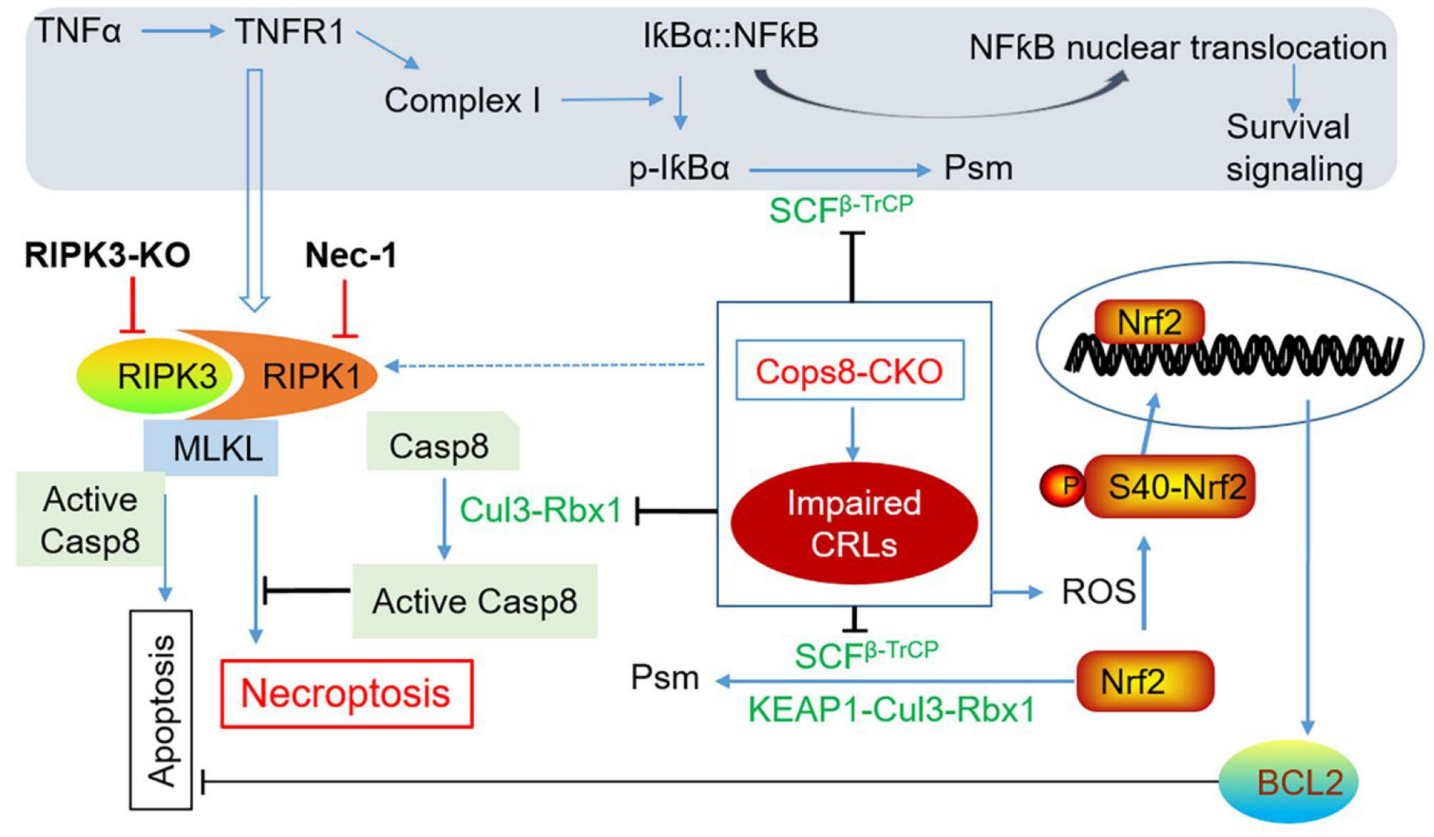
A working model for induction of cardiomyocyte necroptosis by Cops8 deficiency. Loss of function of Cops8/CSN is expected to suppress the CRLs shown in green font, which in turn disrupts the respective ubiquitination events and thereby blocks NFκB-mediated survival signaling and inhibits the apoptotic pathway *via* increasing BCL2 and hindering the activation of caspase-8 (Casp8), steering the death receptor-triggered pathway to the RIPK1–RIPK3-mediated necroptosis. The pathways in the shaded zone are likely involved but not directly examined in Cops8-deficient mice. It is hypothesized that TFNα comes from autocrinal or paracrinal routes from the myocardium with cardiomyocyte-restricted *Cops8* knockout (Cops8-CKO). The interrogations that have been completed are marked with bold black font. Dotted line denotes a potential link that has not been tested yet. Nec-1, necrostatin-1; p-IκBα, phosphorylated IκBα; Psm, proteasomal degradation; RIPK3-KO, global knockout of RIPK3; ROS, reactive oxygen species [adopted from Xiao et al. (2020)].

Second, the release of DAMPs from the necrotic cardiomyocytes can conceivably further induce inflammatory responses that lead to increased secretion of inflammatory cytokines including TNFα, which in turn could trigger the death receptor signaling of the cardiomyocytes *via* endocrinal, paracrinal, or autocrinal modes. As elaborated below, the malfunction of CRLs intrinsic to Cops8 deficiency due to loss of Cullin deneddylation can affect multiple pathways in such a manner that the TNFα-triggered signaling is ultimately steered to the RIPK1–RIPK3 necroptotic pathway in Cops8-deficient cardiomyocytes (Figure 4). As illustrated in Figure 2, the engagement of TNFR1 by TNFα can potentially trigger at least three downstream events: (1) formation of the membrane-associated Complex I where RIPK1 and its ubiquitinated forms serve as a scaffold in a manner independent of its kinase activity, which produces survival signals through activation of NFκB pathway and MAPKs; (2) formation of Complex IIa, which leads to apoptosis *via* caspase-8 and downstream events in the so-called extrinsic apoptotic pathway; and (3) formation of Complex IIb (i.e., the RIPK1–RIPK3–MLKL), thus induction of necroptosis when caspase-8 is absent or suppressed (Del Re et al., 2019). The kinase activity of RIPK1 is indispensable for RIPK1 to mediate programmed cell death in Complex IIa. UPS-dependent degradation of IκBα is an essential step for TNFα to activate NFκB where the K48-linked ubiquitination of IκBα is catalyzed by Skp1-Cul1-β-TrCP (SCF^β−TrCP^) (Kanarek and Ben-Neriah, 2012), a member of the CRL1 family of ubiquitin ligases. Since the catalytic dynamics of CRLs is perturbed by Cops8-cko, it is very likely that the NFκB-centered survival signaling in cardiomyocytes is impaired in Cops8-cko mice. Myocardial F-box protein β-TrCP protein levels were found to be lower in Cops8-cko mice compared with control mice (Su et al., 2011b), arguing further for a predicted reduction of SCF^β−TrCP^ ligase activities. Therefore, there is a great possibility that Cops8 deficiency steers TNFR1 signaling toward cell death direction. It is not known yet whether serum or myocardial TNFα is increased in Cops8-cko mice, and the impact of Cops8 deficiency on the NFκB signaling in cardiomyocytes remains to be determined.

Third, why cardiomyocyte necroptosis rather than apoptosis takes place in Cops8-cko mice? The induction of the necroptotic pathway by death receptor activation has two prerequisites: a) the so-called Complex IIb containing RIPK1 and RIPK3 is formed and b) caspase-8 cannot be activated (Del Re et al., 2019). The study by Xiao et al. (2020) has shown that both prerequisites are met in the Cops8^CKO^ hearts. Myocardial RIPK1, RIPK3, and MLKL protein levels as well as RIPK1-bound RIPK3 were all significantly increased in Cops8-cko mice; and importantly, impaired caspase-8 activation as reflected by markedly decreases in the cleaved form of caspase-8 and in caspase-8 activity were observed in mice with perinatal Cops8^CKO^ compared with mice with control genotypes (Xiao et al., 2020). This caspase-8 impairment is likely due to the loss of Cullin deneddylation because Cul3-RBX1-mediated polyubiquitination of caspase-8 is essential for further processing and activation of caspase-8 and for the progression of the extrinsic apoptotic pathway (Jin et al., 2009). Both neddylation and deneddylation of Cullins are indispensable for the assembly and disassembly of CRLs; hence, the ubiquitination of caspase-8 by Cul3-RBX1 is conceivably compromised by Cops8 deficiency. This postulate is further supported by that inhibition of neddylation with a NEDD8 activation enzyme inhibitor MLN4924 makes monocytes more susceptible to necroptosis *in vitro* (El-Mesery et al., 2015). As discussed in section *The RIPK1–RIPK3–MLKL pathway*, the deubiquitinating enzyme CYLD deubiquitinates RIPK1 to decrease its interaction with NEMO, thus destabilizing Complex I, whereas it also deubiquitinates RIPK1 in TNFα-induced necrosomes to facilitate kinase activation and necroptosis (O'Donnell et al., 2011; Moquin et al., 2013). CYLD was shown to be negatively regulated by caspase-8-mediated cleavage, protecting against TLR-mediated necroptosis (Legarda et al., 2016). Therefore, it is possible that the reduced caspase-8 activation in Cops8-cko hearts promotes cardiomyocyte necroptosis *via* accumulation of CYLD. It will be very interesting to examine whether CYLD is increased in Cops8-deficient cardiomyocytes and whether CYLD plays a critical role in cardiomyocyte cell death caused by Cops8/CSN deficiency.

Not only the extrinsic pathway (as discussed above) but also the intrinsic pathway of apoptosis is likely suppressed in Cops8-cko cardiomyocytes because the powerful anti-apoptotic factor Bcl2 was significantly increased (Su et al., 2011b; Xiao et al., 2020). Increased ROS has been shown to play a role in RIPK3-mediated necroptosis in cultured cells (Zhang et al., 2009; Schenk and Fulda, 2015). In the induction of necroptosis by TNFα, the RIPK3-centered necrosome stimulates aerobic metabolism and thereby increases ROS production; it appears that RIPK3 does so through activation of key metabolic enzymes such as glycogen phosphorylase (PYGL), glutamate-ammonia ligase (GLUL), glutamate dehydrogenase 1 (GLUD1) (Zhang et al., 2009), and more recently pyruvate dehydrogenase (PDH), a rate-limiting enzyme linking glycolysis to aerobic respiration (Yang et al., 2018). The elevated ROS further facilitates the formation of necrosomes and increases cytotoxicity during necroptosis (Schenk and Fulda, 2015). Elevated levels of ROS or oxidative stress are indeed associated with cardiomyocyte necroptosis in Cops8^CKO^ mice, as evidenced by increased levels of protein carbonyls and superoxide anion (${\text{O}}_{2}^{-}$) in Cops8^CKO^ hearts (Xiao et al., 2020). As revealed by the transcriptome analysis (Abdullah et al., 2017), the increased oxidative stress apparently induced the nuclear factor erythroid 2-related factor 2 (Nrf2) pathway, the master regulator of antioxidant and defensive responses, in Cops8^CKO^ hearts even before cardiomyocyte necrosis was discernible (Abdullah et al., 2017). The activation of cardiac Nrf2 by ROS is expected to be more robust in Cops8-cko mice than in WT mice as the UPS-mediated degradation of Nrf2 is likely impaired by Cops8 deficiency. This is because SCF^β−TrCP^ and KEAP1-Cul3-Rbx1, the two known ubiquitin ligases for Nrf2 ubiquitination, are CRLs and expected to be impaired by Cops8/CSN deficiency. The increases in myocardial mRNA levels of Bcl2 (Xiao et al., 2020), a known target gene of Nrf2 (Niture and Jaiswal, 2012), further attest Nrf2 activation in Cops8-cko hearts. Nevertheless, the role of an altered redox state in cardiomyocyte necroptosis induced by Cops8/CSN deficiency has not been established.

## Concluding Remarks and Future Directions

In conclusion, COPS8 and very likely the CSN suppress cardiac RIPK1–RIPK3 necroptotic pathway *in vivo*. The underlying mechanism remains unclear, but the regulatory roles of the COPS8/CSN in both autophagosome maturation and supporting the catalytic dynamics of CRLs *via* Cullin deneddylation are conceivably involved. To establish these conceived molecular links, many questions remain to be addressed. For example, to confirm Cullin deneddylation is required, it will be essential to test whether ablation of other CSN subunits in cardiomyocytes yields the same phenotype as Cops8-cko. The phenotype reported for Cops6-cko shares a lot of similarities with that of Cops8-cko (Liang et al., 2021), but it remains unclear whether cardiomyocyte necrosis occurs in Cops6-cko mice. COPS8/CSN is required for autophagosome maturation in at least cardiomyocytes (Su et al., 2011a), but the molecular underpinning for this requirement remains unknown; it is even unclear whether Cullin deneddylation is a mediating mechanism. To establish the role of decreased autophagic flux or of p62 upregulation will require testing if improving autophagic flux or prevention of p62 accumulation, respectively, can attenuate cardiomyocyte necroptosis in Cops8-cko mice. The regulation of death receptor signaling involves arguably the most diverse forms of ubiquitination (e.g., K48, K63, and linear ubiquitination). They signal for either proteolytic or non-proteolytic fates and are regulated by many ubiquitin ligases (of which some are CRLs), ubiquitin-editing enzymes (e.g., A20), and DUBs (e.g., CYLD, OTULIN); hence, it will be extremely important to understand how COPS8/CSN regulates these regulators and thereby the death receptor signaling. Although the *bona fide* biochemical activity of the CSN is Cullin deneddylation, COPS subunits or minicomplexes of them may also exert deneddylase-independent function. For example, in Cops8 hypomorphic mouse embryonic fibroblasts, the free Cops5 or the Cops5-containing minicomplex may promote the cell cycle transition from G0/G1 to S phase *via* facilitation of nuclear exclusion of p27 and thereby promoting cell proliferation (Liu et al., 2013). Hence, it will also be interesting to investigate the potential involvement of the minicomplex in cardiomyocyte survival. Since both the CSN/Cullin deneddylation and necroptosis have been implicated to play a significant role in cardiac pathogenesis, more intensive effort on deciphering the molecular mechanisms that govern these processes is expected to facilitate the search for new measures to prevent or more effectively treat heart disease.

## Data Availability Statement

The original contributions presented in the study are included in the article, further inquiries can be directed to the corresponding author.

## Author Contributions

ML preparing the first draft of ~15% of the manuscript and preparing Figures 1–3 and their legends under the guidance of XW. TC preparing the first draft of ~10% of the manuscript. XW planning and organizing the overall manuscript, writing ~75% of the manuscript, incorporating various parts into the manuscript, polishing, and corresponding to the editors. All authors have read and approved the manuscript.

## Conflict of Interest

The authors declare that the research was conducted in the absence of any commercial or financial relationships that could be construed as a potential conflict of interest.
